# The Sixty-five Roses of Cystic Fibrosis: A Report of Two Autopsy Cases with Kidney Involvement

**DOI:** 10.7759/cureus.5641

**Published:** 2019-09-13

**Authors:** George S Stoyanov, Hristo Popov, Lilyana Petkova, Deyan L Dzhenkov

**Affiliations:** 1 General and Clinical Pathology, Forensic Medicine and Deontology, Medical University of Varna, Varna, BGR

**Keywords:** cystic fibrosis, kidney, pathology, histology, autopsy

## Abstract

Cystic fibrosis (CF), also commonly referred to as mucoviscidosis, is a multigene related disorder, involving a defect in the CF transmembrane conductance regulator protein, with over 1,500 genes, being identified with the condition. The most commonly affected organs, often described in the literature, are the lungs, pancreas, intestines, and skin, which is one of the sites for early diagnostic testing. Herein we report two autopsy cases of CF, with multiorgan involvement and some rarely observed and reported changes. Two pediatric cases of clinically confirmed CF were referred for autopsy at the Department of General and Clinical Pathology, St. Marina University Hospital, Varna, Bulgaria. The first case was of a one-year-old female and the second of a six-month-old female. Both cases had classical CF-associated changes in the lungs, liver, pancreas, and small intestine. The kidneys although normal on gross inspection also had severe changes on histology with a compacted matter in the lumen of the distal tubules, some of which had undergone calcification. These histological renal changes are under-reported in literature, thus unlike the classical reported cystic changes we highlight lumen compaction and calcification as the primary histological hallmark in kidneys of patients with CF.

## Introduction

Cystic fibrosis (CF), also commonly referred to as mucoviscidosis, is a multigene related disorder, involving a defect in the CF transmembrane conductance regulator (CFTR) protein, with over 1,500 genes, being identified with the condition [1-3]. Defects in the protein cause the production of thick viscous mucus in a number of organs increasing risk of infection and obstructing secretory ducts and canals.

After blockage, the ducts undergo cystic dilation and fibrous tissue develops around them, hence the name CF [4-5]. The most commonly affected organs, often described in the literature, are the lungs, pancreas, intestines, and skin, which is one of the sites for early diagnostic testing [4-5].

Herein we report two autopsy cases of CF, with identical morphology, multiorgan involvement, and some rarely observed and discussed changes.

## Case presentation

Two pediatric cases of clinically confirmed CF were referred for autopsy at the Department of General and Clinical Pathology, St. Marina University Hospital, Varna, Bulgaria.

The first case was of a one-year-old female and the second was of a six-month-old female.

**Figure 1:**
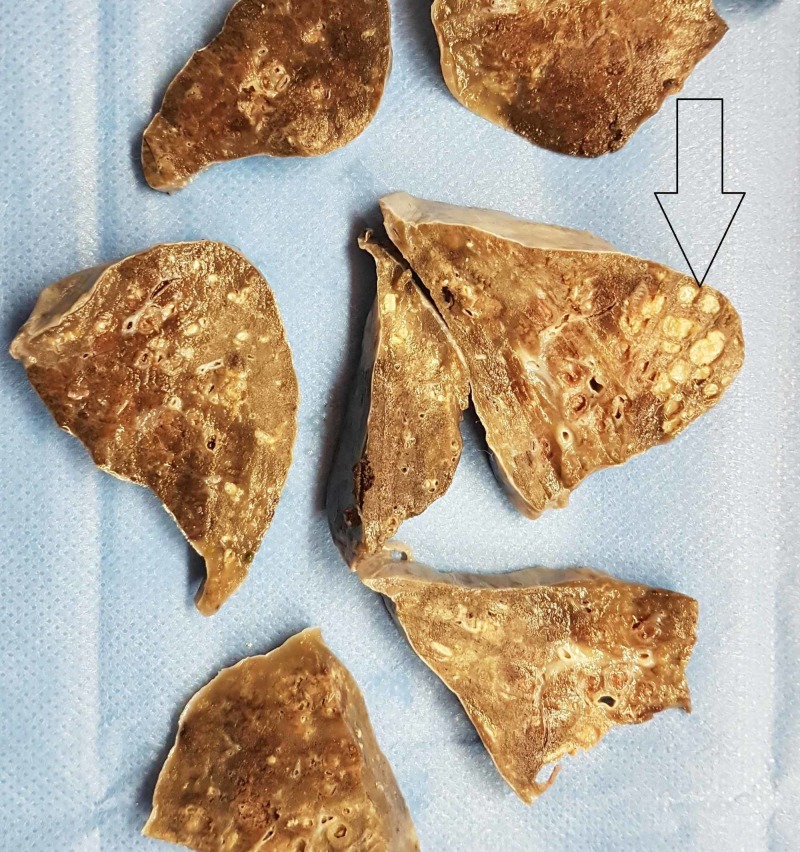
Gross view of the lung after fixation, with well-defined cystic changes (arrow).

**Figure 2:**
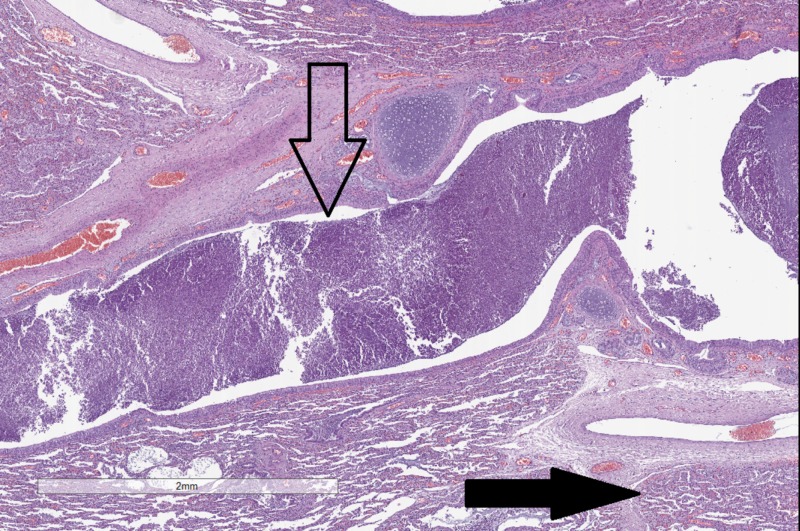
Histopathological changes in the lungs. Cystic bronchial dilation, with a compacted matter in the lumen of the bronchi (hollow arrow) and inflammatory infiltrates in the alveoli (black arrow). Original magnification 20x, hematoxylin and eosin stain.

Both cases had classical CF-associated gross changes in the lungs - cystic dilation of bronchi (up to 1 cm), from which under pressure some thick, pale-yellowish, viscous discharge could be evacuated (Video 1 and Figure 1). On histology, areas with lung parenchymal destruction, profuse polymorphonuclear infiltration, and dilated bronchi filled with slime-like detritus matter were clearly visible across all pulmonary sections (Figure 2).

**Video 1:** Gross view of the lung changes, seen during the autopsy.

**Figure 3:**
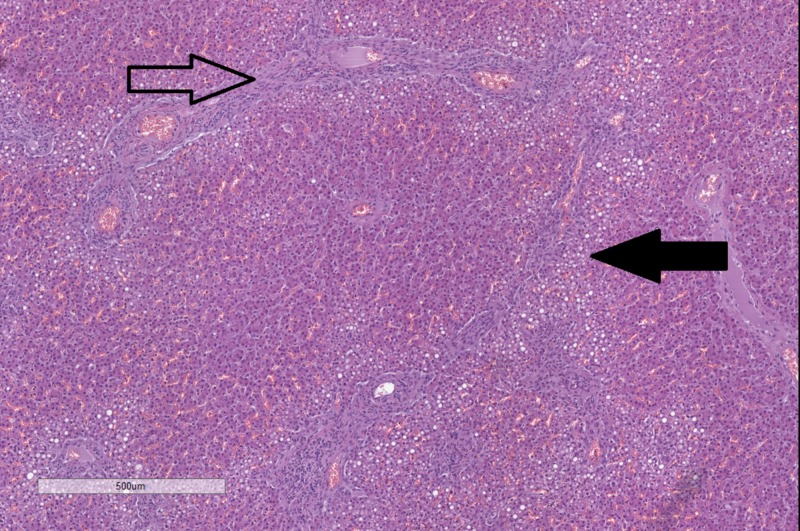
Histopathology of changes in the liver. Fibrosis in the portal areas (hollow arrow) and steatosis (black arrow). Original magnification 50x, hematoxylin and eosin stain.

The liver in both cases was enlarged and yellowish in color, with a distorted architecture of the cut surface. Histology revealed steatosis and cirrhotic changes (Figure 3).

**Figure 4:**
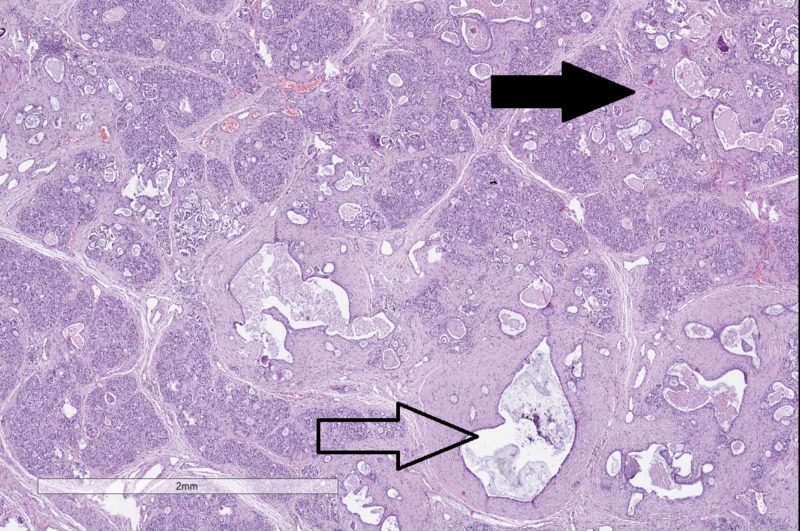
Histopathology of changes in the pancreas. Cystic dilation of the intrapancreatic canals (hollow arrow) and fibrosis (black arrow). Original magnification 20x, hematoxylin and eosin stain.

The pancreas grossly had cystic changes and histology revealed lobularization and fibrosis with the formation of cysts and dilated intrapancreatic ducts (Figure 4).

**Figure 5:**
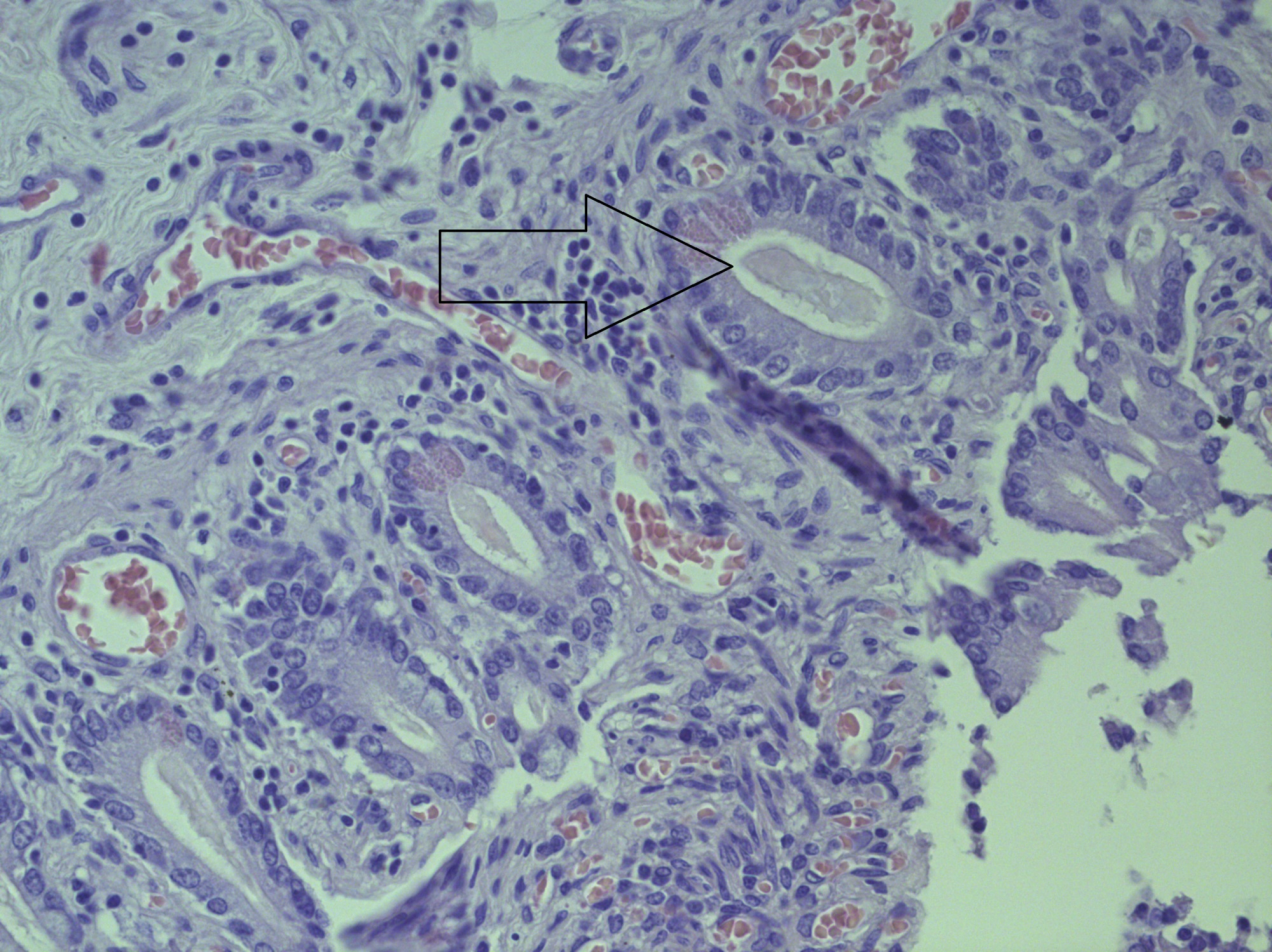
Histopathology of changes in the small intestine. Thick mucus secretions in the glands (arrow). Original magnification 200x, hematoxylin and eosin stain.

Although grossly only hyperemic, the gastrointestinal system revealed surface epithelium erosions and severe inflammatory infiltrates in the mucosa, as well as thick viscous secretions in the glands (Figure 5).

**Figure 6:**
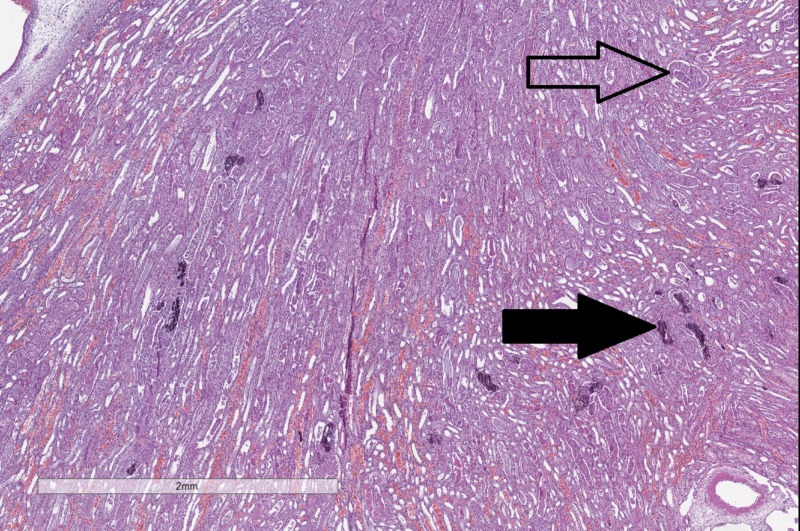
Histopathology of changes in the kidneys. Compacted matter in the distant tubules (hollow arrow) and tubular calcifications (black arrow). Original magnification 20x, hematoxylin and eosin stain.

The kidneys although normal on gross inspection also had severe changes on histology with a compacted matter in the lumen of the distal tubules, some of which had undergone calcification (Figure 6).

The cause of death in both cases was related to complications of CF, due to lung, liver, pancreas, intestinal, and renal involvement.

## Discussion

The international symbol of CF are the sixty-five roses, due to the similarity in pronunciation. However, of the six organ locations affected - lungs, liver, pancreas, intestines, skin, and kidneys, only five are discussed in depth in the scientific literature [1-4].

The renal changes may trigger additional complications and exacerbate the course of the condition if misdiagnosed and untreated. Still, the renal changes, although often cited are poorly defined in the literature, with the predominant dogma being that they also undergo cystic change, similar to polycystic kidney disease [6-7].

As seen in our two cases, the changes, at least in the initial stages of the evolution of CF, do not result in cystic degeneration, but rather in distant tubule compaction and calcification, which with time may result in kidney failure, exacerbating the overall condition of the patients.

The identical morphology, present in both of the cases, across all organ systems, is key in defining the renal changes, not as incidental, but as associated with the pathogenesis of CF, albeit in an early stage. These changes, may in time progress to the proposed polycystic-like morphology or be an all separate morphological substrate of CF. Also worth noting is that these changes go hand-in-hand with other organ changes, as seen by the age of both patients, and do not represent a late complication of CF. However, the changes clinically may present as a late complication, often attributed to multiorgan failure.

This with time and especially in patients where the overall condition is stable and susceptible to therapy, the described changes may lead to chronic kidney failure as the leading cause of complications and hospital stay in CF.

## Conclusions

Cystic fibrosis is a relatively common genetic disorder, with a wide spectrum of organ changes. If misdiagnosed and untreated, these changes may contribute to severe complications. These histological renal changes are under-reported in literature, thus unlike the classical reported cystic changes we highlight lumen compaction and calcification as the primary histological hallmark in kidneys of patients with CF.
